# Biweekly fluctuations of neuropsychiatric symptoms according to the Neuropsychiatric Inventory: Erratic symptoms or scores?

**DOI:** 10.1002/gps.5770

**Published:** 2022-06-15

**Authors:** Willem S. Eikelboom, Amy den Teuling, Daphne E. Pol, Michiel Coesmans, Sanne Franzen, Lize C. Jiskoot, Judy van Hemmen, Ellen H. Singleton, Rik Ossenkoppele, Frank Jan de Jong, Esther van den Berg, Janne M. Papma

**Affiliations:** ^1^ Department of Neurology and Alzheimer Center Erasmus MC Erasmus MC University Medical Center Rotterdam The Netherlands; ^2^ Department of Psychiatry Erasmus MC University Medical Center Rotterdam The Netherlands; ^3^ Department of Neurology Alzheimer Center Amsterdam Amsterdam University Medical Centers Amsterdam The Netherlands; ^4^ Clinical Memory Research Unit Lund University Malmö Sweden

**Keywords:** Alzheimer's disease, behavioral and psychological symptoms of dementia (BPSD), behavioral symptoms, dementia, Neuropsychiatric Inventory, neuropsychiatric symptoms

## Abstract

**Objectives:**

This study investigates the stability of neuropsychiatric symptoms (NPS) assessed biweekly using the Neuropsychiatric Inventory (NPI) in a memory clinic population during a 6 week period.

**Methods:**

Twenty‐three spousal caregivers (mean [SD] age = 69.7 [8.8], 82.6% female) of 23 patients (43.5% had dementia) completed all assessments. The NPI was assessed four times during 6 weeks. We examined whether NPI domains were present during all four assessments, studied within‐person variation for each NPI domain, and calculated Spearman's correlations between subsequent time‐points. Furthermore, we associated repeated NPI assessments with repeated measures of caregiver burden to examine the clinical impact of changes in NPI scores over time.

**Results:**

The course of NPS was highly irregular according to the NPI, with only 35.8% of the NPI domains that were present at baseline persisted during all 6 weeks. We observed large within‐person variation in the presence of individual NPI domains (61.3%, range 37.5%–83.9%) and inconsistent correlations between NPI assessments (e.g., range *r*_*s*_ = 0.20–0.57 for agitation, range *r*_*s*_ = 0.29–0.59 for anxiety). Higher NPI total scores were related to higher caregiver burden (*r*_*s*_ = 0.60, *p* < 0.001), but changes in NPI total scores were unrelated to changes in caregiver burden (*r*_*s*_ = 0.16, *p* = 0.20).

**Conclusions:**

We observed strong fluctuations in NPI scores within very short time windows raising the question whether this represents erratic symptoms and/or scores. Further studies are needed to investigate the origins of these fluctuations.

## Key points

- The presence and severity of all neuropsychiatric symptoms (NPS) measured with the Neuropsychiatric Inventory (NPI) were highly variable within a memory clinic population when repeatedly assessed during 6 weeks.
- Although we found subtle differences between longitudinal NPI domain scores, not a single domain score was stable over time.
- Findings either reflects the fluctuating nature of NPS in neurocognitive disorders and/or the limited ability of the NPI to reliably measure NPS in a memory clinic population.

## 1 INTRODUCTION

Neuropsychiatric symptoms (NPS) such as depression, apathy, agitation, and sleep disturbances are frequently observed in individuals who visit the memory clinic.^1^, ^2^ These symptoms have a major impact on the lives of patients and their caregivers and are associated with increased caregiver burden.^3^, ^4^, ^5^ The Neuropsychiatric Inventory (NPI) is considered the gold standard to assess NPS in neurocognitive disorders.^6^

Previous studies that examined the course of NPS using the NPI have shown large within‐person variability in the progression of NPI scores when administering the NPI every 6–12 months.^1^, ^7^, ^8^, ^9^ It remains unclear whether there is also such within‐person heterogeneity in longitudinal NPI scores when measured during shorter time intervals, for example, within weeks instead of months.^7^ Although several studies have administered the NPI twice within a timeframe of 2–3 weeks to establish the test‐retest reliability of the NPI,^10^ knowledge on short‐term trajectories of NPS according to repeated NPI assessments is lacking.

Here, we describe the stability of NPI scores over a period of 6 weeks in a memory clinic population. During this 6 week period, the NPI was administered biweekly in order to compare our findings with previous test‐retest studies that have assessed the NPI within a similar timeframe.^11^, ^12^, ^13^ Furthermore, we compared the trajectories of NPI scores with repeated measures of caregiver burden. NPS is a well‐known contributor to caregiver burden.^3^, ^4^, ^5^ Therefore, we included a measure of caregiver burden to examine the clinical impact of short‐term changes in NPI scores. Based on previous test‐retest studies,^10^ we hypothesized stable NPI scores over time for apathy and psychotic symptoms, while we expected less stable NPI scores for affective symptoms, agitation‐related behaviors, and sleep disturbances.

## 2 METHODS

### 2.1 Study design and participants

We invited all caregivers of patients who visited the memory clinic of the Erasmus MC in Rotterdam, the Netherlands, between June 2020 and July 2020, and between November 2020 and January 2021, to participate in this study. We included participants regardless of clinical diagnosis and presence/severity of NPS at baseline, with the only requirement that caregivers had to live with the patient. All patients underwent a standard diagnostic workup including medical history taking, neurological examination, neuropsychological assessment, and brain MRI. Clinical diagnoses were established using conventional diagnostic criteria during a multidisciplinary meeting.

### 2.2 Measures

The Dutch NPI and Dutch Caregiver Strain Index (CSI)‐Expanded were administered in person to caregivers during the initial the memory clinic visit.^14^, ^15^ During the 6 weeks that followed, the NPI and CSI‐Expanded assessments were repeated every 2 weeks by telephone. Caregivers evaluated the presence, frequency (0–4), severity (0–3), and distress (0–5) of NPS in the previous 2 weeks. NPI domain scores were calculated by multiplying the frequency and severity scores (0–12). The presence of specific NPI domains was defined as an NPI domain score of ≥1. We summed the 12 NPI domain scores to obtain the NPI total score (0–144).^14^ The Dutch CSI‐Expanded was used to assess caregiver burden. This instrument covers aspects of caregiver strain (13 items) and aspects of caregiving that may decrease burden (5 items), resulting in a total score ranging between −5 and 13.^15^

### 2.3 Data analysis

We examined the prevalence of specific NPI domains at baseline and its persistence. NPI domains were persistent if they were present on all four assessments. For each NPI domain, we described the between‐person variation (i.e., how many individuals had an NPI domain score of ≥1 at least once) and the within‐person variation (i.e., total number of assessments in which NPS were present in individuals who had an NPI domain score of ≥1 at least once, with both 0% and 100% indicating no variation).^8^ For each NPI domain, we conducted Spearman's correlations to examine the relationship between NPI domain scores on subsequent time‐points (baseline‐t1, t1–t2, t2–t3). Individual trajectories of NPI domain scores over time were plotted for descriptive purposes, but not analyzed at group‐level.

We correlated NPI total scores with CSI‐Expanded total scores across all time‐points. Next, we calculated delta scores for NPI total scores and CSI‐Expanded total scores for each time‐point and associated these delta scores using Spearman's correlations.

To examine the effects of cognitive status, we conducted exploratory analysis in which prevalence, persistence, between‐person variation, within‐person variation, and Spearman's correlations between NPI domain scores were performed for patients with dementia and patients with cognitive impairment no dementia (CIND) separately.

### 2.4 Ethics

This study received ethical approval from the Medical Ethics Committee of the Erasmus University Medical Center (MEC‐2020‐0341). All participants gave informed consent.

## 3 RESULTS

### 3.1 Study participants

A total of 26 caregivers agreed to participate in this study. There were three drop‐outs during the study due to perceived burden (*n* = 1), acute health problems of the caregiver (*n* = 1), and loss of contact (*n* = 1). All analyses were conducted in the 23 caregivers who completed all assessments (Table 1). Caregivers had a mean age of 69.7 (SD = 8.8), 82.6% were female, and all were spouses. The patients had a mean age of 72.8 (SD = 8.2) and 21.7% were women. Most patients were diagnosed with dementia (*n* = 10, 43.5%), eight individuals had CIND (34.8%), and four patients had no evidence of cognitive impairment (17.4%). A clinical diagnosis could not be determined in one individual (4.3%). Two patients (8.7%) were on a stable dose of psychotropic medications, while Escitalopram was prescribed during study period in only one patient (4.3%). A cognitive enhancer was prescribed during the 6 week period for two patients (8.7%).

**TABLE 1:** Baseline characteristics of included sample

Caregivers (*n* = 23)	
Age, median (IQR)	71.0 (7.0)
Sex, *N* female (%)	18 (78.3%)
Education, median (IQR)	5.0 (2.0)
Relationship with patient, *N* (%)	
Spouse	23 (100.0%)

Patients (*n* = 23)	
Age, median (IQR)	74.0 (11.0)
Sex, *N* female (%)	5 (21.7%)
Education, median (IQR)	5.0 (2.0)
Clinical diagnosis, *N* (%)	
Dementia	10 (43.5%)
Alzheimer's disease dementia	6
Primary Progressive Aphasia	2
Behavioral variant frontotemporal dementia	1
Corticobasal syndrome	1
Cognitive impairment no dementia	8 (34.8%)
Mild cognitive impairment	6
Radiation‐induced cognitive decline	1
Cognitive impairment due to epilepsy	1
No cognitive impairment	4 (17.4)
Subjective cognitive decline	3
Major depressive episode	1
Could not be determined	1 (4.3%)
Months since clinical diagnosis, median (IQR)^a^	0.0 (0.0)
Mini‐mental state examination score, median (IQR)^b^	26.0 (9.0)
Cognitive enhancer use at baseline, *N* (%)	0 (0.0%)
Cognitive enhancer started during study, *N* (%)	2 (8.7%)
Psychotropic drug use at baseline, *N* (%)	2 (8.7%)
Psychotropic drug started during study, *N* (%)	1 (4.3%)

Abbreviation: IQR, interquartile range.

^a^ Not applicable for *n* = 1.

^b^ Missing data for *n* = 3.

### 3.2 Prevalence and course of NPS according to the NPI

At baseline, all caregivers indicated the presence of at least one NPS [mean number of NPI domains was 3.0 (range 1–6)], with a mean NPI total score of 12.3 (SD = 9.5). Irritability (56.5%), sleep disturbances (47.8%), and depression (42.3%) were most common at baseline (Table 2). Across all NPI domains, the within‐person variation was 61.3% (range 37.5%–83.9%), indicating that NPI domains that were present once during the course of the study were observed at 61.3% of the four time‐points (Table 2). Only 35.8% (range 0.0%–100.0%) of the NPI domains that were present at baseline persisted over all three follow‐up assessments (Table 1). There were no substantial differences between patients with dementia and CIND in within‐person variation across NPI domains [dementia: 63.5% (range 25.0%–94.3%), CIND: 62.8% (range 25.0%–79.2%)] and persistence of NPI domains (dementia: 37.2%, CIND: 35.8%) (see Table S2).

**TABLE 2:** Prevalence, persistence, and between‐person and within‐person variation of the presence of specific NPI domains

NPI domain	Presence at baseline	Persistence^a^	Between‐person variation^b^	Within‐person variation^c^
Irritability	13 (56.5%)	7 (53.8%)	18 (78.3%)	70.8%
Sleep disturbances	11 (47.8%)	8 (72.7%)	14 (60.9%)	83.9%
Depression	10 (42.3%)	3 (30.0%)	16 (69.6%)	65.5%
Apathy	8 (34.8%)	4 (50.0%)	12 (52.2%)	60.4%
Anxiety	8 (34.8%)	1 (12.5%)	9 (39.1%)	55.6%
Eating behavior	6 (26.1%)	3 (50.0%)	10 (43.5%)	62.5%
Agitation	5 (21.7%)	1 (20.0%)	9 (39.1%)	61.1%
Aberrant motor behavior	5 (21.7%)	2 (40.0%)	7 (30.4%)	71.4%
Disinhibition	1 (4.3%)	0 (0.0%)	6 (26.1%)	50.0%
Euphoria	1 (4.3%)	1 (100.0%)	3 (13.0%)	50.0%
Hallucinations	1 (4.3%)	0 (0.0%)	4 (17.4%)	37.5%
Delusions	1 (4.3%)	0 (0.0%)	3 (13.0%)	66.7%

Abbreviations: NPI, Neuropsychiatric Inventory; NPS, neuropsychiatric symptoms.

^a^ *N* (%) of individuals which showed NPS during all follow‐up assessments when present at baseline.

^b^ *N* (%) of individuals with NPS present at least at one time‐point.

^c^ For those with NPS at one time‐point, % of assessments NPS was present.

**FIGURE 1:**
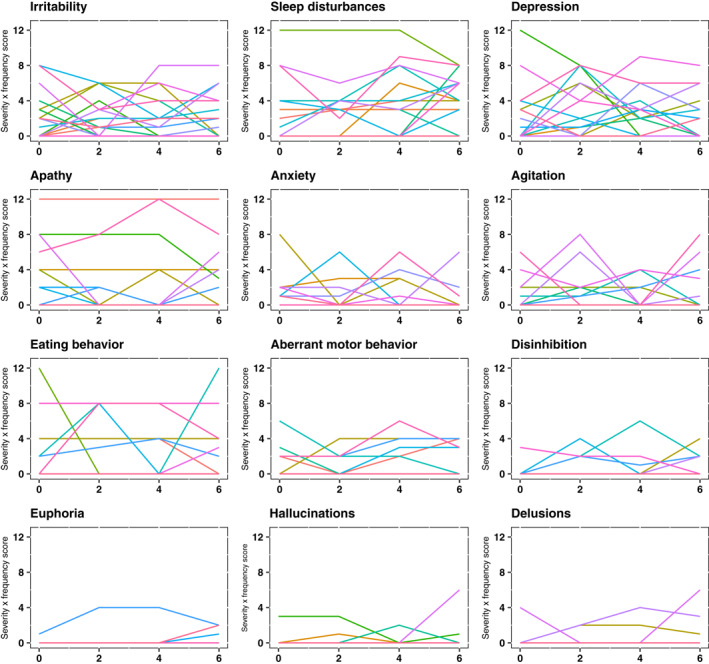
NPI domain scores assessed biweekly. Each line represents the trajectories of the severity × frequency score (0–12) of each NPI domain for an individual participant assessed every 2 weeks during a 6 week period. NPI, Neuropsychiatric Inventory

Figure 1 shows considerable heterogeneity in course of NPI domain scores between individuals, but especially reveals substantial fluctuations within individuals. Spearman's correlations between NPI domain scores at two subsequent time‐points (baseline‐t1, t1–t2, t2–t3) varied greatly within NPI domains (see Table S1). NPI total scores correlated significantly between time‐points (range *r*_*s*_ = 0.55–0.67, *p* < 0.01), while low and inconsistent correlation coefficients were observed for specific NPI domains such as agitation (range *r*_*s*_ = 0.20–0.57), irritability (range *r*_*s*_ = 0.26–0.65), aberrant motor behavior (range *r*_*s*_ = 0.55–0.90), and anxiety (range *r*_*s*_ = 0.29–0.59). Spearman's correlations were slightly higher in patients with dementia compared to individuals with CIND for NPI total scores (dementia: range *r*_*s*_ = 0.70–0.87, all *p* < 0.05, CIND range *r*_*s*_ = 0.56–0.83, 2/3 *p* > 0.05) and several NPI domain scores (see Table S3).

We considered the presence of sleep disturbances, irritability, and aberrant motor behavior to be most stable, while the presence of hallucinations, disinhibition, and anxiety were the least stable within persons (Table 2). When present, we considered the severity/frequency of apathy, sleep disturbances, and euphoria most stable, while depression, anxiety, and hallucinations were the least stable (Figure 1 and Table S1).

### 3.3 Data associations between NPI scores and caregiver burden

Across all time‐points, higher NPI total scores were related to higher caregiver burden (*r*_*s*_ = 0.60, *p* < 0.001). However, changes in NPI total scores were unrelated to changes in caregiver burden (*r*_*s*_ = 0.16, *p* = 0.20).

## 4 DISCUSSION

This study shows that NPI scores at one time‐point in a memory clinic sample are poorly related to NPI scores administered only 2 weeks later. Our findings provide further evidence for the large variability of NPI scores within individuals with neurocognitive disorders.^1^, ^7^, ^8^, ^9^ When looking at specific NPI domains, we found lowest stability over time for anxiety, hallucinations, depression, and disinhibition, which is in line with prior test‐retest studies.^10^, ^11^, ^12^, ^13^ Our findings extend previous studies by looking at trajectories over a period over several weeks compared to the commonly examined (bi)annual NPI assessments.^1^, ^7^, ^8^, ^9^

The large within‐person variation in NPI scores could reflect substantial fluctuations in the manifestation of NPS in patients visiting the memory clinic. Previous studies that have used diaries to daily assess NPS in dementia suggested a rather erratic nature of NPS.^16^, ^17^, ^18^ This is in line with the growing body of evidence emphasizing the role of proximal causes of NPS including psychosocial factors (e.g., caregiver burden, caregiver communication style), environmental factors (e.g., light, temperature), and somatic conditions (e.g., pain, thirst).^17^, ^19^

Alternatively, the irregular course of NPI scores could also arise from methodologic issues related to the NPI. Our finding that changes in NPI scores were unrelated to changes in caregiver burden could support this. Several factors could affect the NPI scores that are unrelated to the actual manifestation of NPS in our sample. First, caregivers tend to use different terminologies to describe NPS compared to the terms used in instruments such as the NPI.^20^ They are also inclined to use broad terms covering multiple NPS that would generally be considered clinically distinct symptoms.^20^ Consequently, caregivers may have endorsed different NPI domains during follow‐up assessments, although similar NPS were present during the course of the study. Furthermore, although recall bias was reduced because caregivers were asked to evaluate the presence of NPS during the last 2 weeks instead of the commonly used 4 weeks, the recollection of NPS remains challenging.^21^ Moreover, mood, fatigue, and distress among caregivers can affect the NPI administration.^21^, ^22^ To overcome these challenges, future studies could pair repeated NPI assessments with daily NPS measurements using an Ecological Momentary Assessments approach.^23^

Also, the variation in NPI scores could be an effect of unknown measurement error related to the NPI as little is known about what we should consider as actual change in NPI scores. Different statistical methods such as the standard error of measurement and the reliable change index have been developed to determine the minimal detectable change of clinical outcome scales.^24^, ^25^ These methods have been used to establish minimal detectable change for the individual domains of the nursing home version of the NPI after 2 weeks and the NPI total score after 52 weeks.^13^, ^26^ However, these psychometric indices establish minimal detectable change and do not determine minimally important change, that is, clinically meaningful change.^24^, ^25^ Anchor‐based approaches can be used to determine clinically meaningful change by which changes on an instrument are compared with minimally important changed defined by patients, caregiver, and/or clinicians. Future studies are needed that align NPI trajectories with anchor definitions of meaningful change in NPS to establish which changes in NPI scores we should consider as clinically meaningful.

### 4.1 Strengths and limitations

Strengths of this study are the inclusion of a representative tertiary memory clinic sample consisting of various clinical diagnoses and the low level of psychotropic medications used across patients. There are also some limitations to our study. First, the majority of the participants were still undergoing diagnostic workup and received a diagnosis at some point during the study. This may have affected the manifestation of NPS as receiving a diagnosis can have great psychological impact. Second, we included a small and clinically heterogeneous memory clinic population. The low sample size may affected the stability of correlation coefficients, especially the correlations below 0.50.^27^ Furthermore, the proportion of female patients (22%) in our sample was lower than expected based on previous studies in Dutch academic memory clinics (40%–55% females).^1^, ^28^, ^29^ Although our within‐person analysis reduces the potential impact of the clinical heterogeneity and underrepresentation of female patients, our results need to be replicated in larger samples including a higher proportion of females, especially since NPS may manifest differently in females than males.^30^ Third, we found indications that NPI scores were somewhat more stable in individuals with dementia compared to individuals with CIND. This suggest that the NPI may be more appropriate to repeatedly assess NPS when used in individuals with dementia, which could be expected as the NPI was originally developed and validated to measure NPS in dementia.^31^ Yet, future studies with larger samples are needed to examine the effects of demographic characteristics and clinical characteristics such as dementia type on the short term trajectories of NPI scores. Finally, no clear cutoffs exist for measures used in this study (e.g., within‐person variation) making the comparison between NPI domains somewhat subjective.

### 4.2 Conclusions

This study suggest highly unstable NPI scores when assessed at 2‐week intervals. These findings question the reliability of NPI scores when administered at short‐term intervals at the memory clinic, but also as outcome measure in trails that evaluate the effectiveness of (non)pharmacological interventions, especially for those who do not meet diagnostic criteria for dementia (i.e., CIND). Further studies are needed to investigate whether the large within‐person variability of NPI scores reflect the erratic nature of NPS in neurocognitive disorders or arise from methodological issues. Although the origin of these fluctuations remains unclear, memory clinic clinicians should be aware that NPI scores at one time point are poorly related to future NPI scores within a timeframe of weeks.

## AUTHOR CONTRIBUTIONS

Willem S. Eikelboom designed the study, analyzed the data, and wrote the paper. Amy den Teuling collected the data and assisted with writing the paper. Daphne E. Pol collected the data and assisted with writing the paper. Michiel Coesmans assisted with writing the paper. Sanne Franzen collected the data and assisted with writing the paper. Lize C. Jiskoot collected the data and assisted with writing the paper. Judy van Hemmen collected the data and assisted with writing the paper. Ellen Singleton assisted with writing the paper. Rik Ossenkoppele assisted with writing the paper. Frank Jan de Jong assisted with writing the paper. Esther van den Berg designed the study and assisted with writing the paper. Janne M. Papma designed the study, supervised the study, and assisted with writing the paper.

## CONFLICT OF INTEREST

The authors declared that they have no conflicts of interest to this work.

## ETHICS STATEMENT

This study received ethical approval from the Medical Ethics Committee of the Erasmus University Medical Center (MEC‐2020‐0341). All participants gave informed consent.

## Supporting information

Supporting Information 1

## Data Availability

The data that support the findings of this study are available on request from the corresponding author. The data are not publicly available due to privacy or ethical restrictions.
