# Supplementary material for: Biweekly fluctuations of neuropsychiatric symptoms according to the Neuropsychiatric Inventory: Erratic symptoms or scores?
Source: Int J Geriatr Psychiatry. 2022 Jun 15;37(7):10.1002/gps.5770. doi: 10.1002/gps.5770 (PMC9327507; doi:10.1002/gps.5770)
Supplement: Supplementary file 1 — Supporting Information 1 [file GPS-37-0-s001.docx]

| **Supplemental Table 1. Spearman’s correlations between NPI domain scores measured at biweekly intervals.** | | | |
| --- | --- | --- | --- |
| **NPI Domain** | **Baseline–Follow-up 1** | **Follow-up 1–Follow-up 2** | **Follow-up 2–Follow-up 3** |
| Aberrant motor behavior | 0.55** | 0.82*** | 0.90*** |
| Agitation | 0.53** | 0.57** | 0.20 |
| Anxiety | 0.59** | 0.29 | 0.47* |
| Apathy | 0.68*** | 0.76*** | 0.47* |
| Delusions | -0.07 | 0.99*** | 0.76*** |
| Depression | 0.39 | 0.49* | 0.64** |
| Disinhibition | 0.44* | 0.80*** | 0.47* |
| Eating behavior | 0.47* | 0.64** | 0.64** |
| Euphoria | 1.00*** | 1.00*** | 0.58** |
| Hallucinations | 0.72*** | -0.07 | -0.07 |
| Irritability | 0.26 | 0.62** | 0.65** |
| Sleep behavior | 0.82*** | 0.73*** | 0.67** |
| NPI total score | 0.55** | 0.59** | 0.67*** |
| *Notes:* * *p* < 0.05, ** *p* < 0.01, *** *p* < 0.001. | | | |

| **Supplemental Table 2. Prevalence, persistence, and between-person and within-person variation of the presence of specific NPI domains.** | | | | | | | | |
| --- | --- | --- | --- | --- | --- | --- | --- | --- |
| **NPI domain** | **Presence at baseline**  **dementia (n=10)** | **Presence at baseline**  **CIND**  **(n=8)** | **Persistence^†^**  **dementia (n=10)** | **Persistence^†^**  **CIND**  **(n=8)** | **BP variation^‡^**  **dementia (n=10)** | **BP variation^‡^**  **CIND**  **(n=8)** | **WP variation^§^**  **dementia (n=10)** | **WP variation^§^**  **CIND**  **(n=8)** |
| AMB | 4 (40.0%) | 1 (12.5%) | 1 (25.0%) | 1 (100.0%) | 4 (40.0%) | 3 (37.5%) | 68.8% | 75.0% |
| Agitation | 2 (20.0%) | 3 (37.5%) | 1 (50.0%) | 0 (0.0%) | 3 (30.0%) | 3 (37.5%) | 83.3% | 66.7% |
| Anxiety | 4 (40.0%) | 3 (37.5%) | 1 (25.0%) | 0 (0.0%) | 4 (40.0%) | 5 (50.0%) | 50.0% | 56.3% |
| Apathy | 3 (30.0%) | 4 (50.0%) | 2 (66.7%) | 1 (25.0%) | 5 (50.0%) | 5 (50.0%) | 65.0% | 55.0% |
| Delusions | 0 (0.0%) | 1 (12.5%) | - | 1 (12.5%) | 0 (0.0%) | 2 (25.0%) | - | 75.0% |
| Depression | 5 (50.0%) | 3 (37.5%) | 1 (20.0%) | 2 (66.7%) | 6 (60.0%) | 6 (75.0%) | 62.5% | 66.7% |
| Disinhibition | 0 (0.0%) | 1 (12.5%) | 0 (0.0%) | 0 (0.0%) | 2 (20.0%) | 3 (37.5%) | 75.0% | 41.7% |
| Eating behavior | 4 (40.0%) | 2 (25.0%) | 1 (25.0%) | 2 (100.0%) | 5 (50.0%) | 4 (50.0%) | 60.0% | 75.0% |
| Euphoria | 1 (10.0%) | 0 (0.0%) | 1 (100.0%) | - | 3 (30.0%) | 0 (0.0%) | 50.0% | - |
| Hallucinations | 0 (0.0%) | 0 (0.0%) | 0 (0.0%) | 0 (0.0%) | 1 (10.0%) | 2 (25.0%) | 25.0% | 25.0% |
| Irritability | 5 (50.0%) | 6 (75.0%) | 3 (60.0%) | 3 (50.0%) | 7 (70.0%) | 6 (75.0%) | 64.3% | 79.2% |
| Sleep behavior | 4 (40.0%) | 4 (50.0%) | 3 (75.0%) | 3 (75.0%) | 4 (40.0%) | 6 (75.0%) | 93.8% | 75.0% |
| *Notes:* AMB = aberrant motor behavior, CIND = cognitive impairment no dementia, BP = between-person, WP = within-person.  **^†^** N (%) of individuals which showed NPS during all follow-up assessments when present at baseline.  **^‡^** N (%) of individuals with NPS present at least at one time-point.  **^§^** For those with NPS at one time-point, % of assessments NPS was present. | | | | | | | | |

| **Supplemental Table 3. Spearman’s correlations between NPI domain scores measured at biweekly intervals.** | | | | | | |
| --- | --- | --- | --- | --- | --- | --- |
| **NPI Domain** | **BL–FU 1**  **dementia (n=10)** | **BL–FU 1**  **CIND (n=8)** | **FU 1–FU 2**  **dementia (n=10)** | **FU 1–FU 2**  **CIND (n=8)** | **FU 2–FU 3**  **dementia (n=10)** | **FU 2–FU 3**  **CIND (n=8)** |
| AMB | 0.64* | 0.54 | 0.81** | 0.84** | 0.81** | 0.95** |
| Agitation | 0.82** | 0.58 | 0.99** | 0.54 | 0.68* | - |
| Anxiety | 0.33 | 0.55 | 0.75* | 0.01 | 0.75* | 0.67 |
| Apathy | 0.86** | 0.44 | 0.79** | 0.76* | 0.75* | 0.29 |
| Delusions | - | -0.14 | - | 1.00** | - | 0.54 |
| Depression | 0.26 | 0.72* | 0.76* | 0.51 | 0.72* | 0.57 |
| Disinhibition | - | 0.54 | 0.99** | 0.54 | 0.99** | -0.14 |
| Eating behavior | 0.63* | 0.44 | 0.64* | 0.72* | 0.11 | 0.98** |
| Euphoria | 0.00 | - | 1.00** | - | 0.57 | - |
| Hallucinations | - | - | - | - | - | - |
| Irritability | 0.28 | 0.38 | 0.89** | 0.43 | 0.65* | 0.61 |
| Sleep behavior | 1.00** | 0.88** | 0.97** | 0.48 | 0.91** | 0.73* |
| NPI total score | 0.70* | 0.83* | 0.86** | 0.56 | 0.87** | 0.69 |
| *Notes:* AMB = aberrant motor behavior, BL = baseline, CIND = cognitive impairment no dementia, FU = follow-up.  * *p* < 0.05, ** *p* < 0.01, *** *p* < 0.001. | | | | | | |
